# Second messenger signalling bypasses CGRP receptor blockade to provoke migraine attacks in humans

**DOI:** 10.1093/brain/awad261

**Published:** 2023-08-04

**Authors:** Thien Phu Do, Christina Deligianni, Sarkhan Amirguliyev, Josefin Snellman, Cristina Lopez Lopez, Mohammad Al-Mahdi Al-Karagholi, Song Guo, Messoud Ashina

**Affiliations:** Department of Neurology, Danish Headache Center, Copenhagen University Hospital—Rigshospitalet, Copenhagen, Denmark; Department of Clinical Medicine, University of Copenhagen, Copenhagen, Denmark; Department of Neurology, Danish Headache Center, Copenhagen University Hospital—Rigshospitalet, Copenhagen, Denmark; Department of Neurology, Danish Headache Center, Copenhagen University Hospital—Rigshospitalet, Copenhagen, Denmark; Novartis Pharma AG, 4056 Basel, Switzerland; Roche Innovation Center Basel, F. Hoffmann-La Roche Ltd., 4070 Basel, Switzerland; Department of Neurology, Danish Headache Center, Copenhagen University Hospital—Rigshospitalet, Copenhagen, Denmark; Department of Neurology, Danish Headache Center, Copenhagen University Hospital—Rigshospitalet, Copenhagen, Denmark; Department of Neurology, Danish Headache Center, Copenhagen University Hospital—Rigshospitalet, Copenhagen, Denmark; Department of Clinical Medicine, University of Copenhagen, Copenhagen, Denmark

**Keywords:** calcitonin, migraine model, cranial arteries, vasodilation, PACAP

## Abstract

There are several endogenous molecules that can trigger migraine attacks when administered to humans. Notably, calcitonin gene-related peptide (CGRP) has been identified as a key player in a signalling cascade involved in migraine attacks, acting through the second messenger cyclic adenosine monophosphate (cAMP) in various cells, including intracranial vascular smooth muscle cells. However, it remains unclear whether intracellular cAMP signalling requires CGRP receptor activation during a migraine attack in humans. To address this question, we conducted a randomized, double-blind, placebo-controlled, parallel trial using a human provocation model involving the administration of CGRP and cilostazol in individuals with migraine pretreated with erenumab or placebo. Our study revealed that migraine attacks can be provoked in patients by cAMP-mediated mechanisms using cilostazol, even when the CGRP receptor is blocked by erenumab. Furthermore, the dilation of cranial arteries induced by cilostazol was not influenced by the CGRP receptor blockade. These findings provide clinical evidence that cAMP-evoked migraine attacks do not require CGRP receptor activation. This discovery opens up new possibilities for the development of mechanism-based drugs for the treatment of migraine.

## Teaser

Using a human migraine provocation model, Do *et al.* provide clinical evidence that cAMP-evoked migraine attacks do not require CGRP receptor activation. These findings open up new avenues for mechanism-based drug development for migraine.

See Levy (https://doi.org/10.1093/brain/awad390) for a scientific commentary on this article.

## Introduction

Migraine is a highly prevalent disease affecting more than 1 billion people worldwide.^1,2^ It is characterized by stereotypical attacks of headache accompanied by nausea, vomiting and hypersensitivity to light and sound.^3,4^ The pathogenesis of migraine attacks involves complex interactions between signalling events within intracranial blood vessels and perivascular trigeminal sensory afferents, denoted as the trigeminovascular system.^3,5^

A key feature of migraine is that various environmental and endogenous trigger factors can initiate an attack.^6^ Given that migraine attacks are self-limiting and treatable, this feature provides a unique opportunity to dissect the underlying molecular signalling cascade *in vivo* in patients. By administering signalling molecules to patients and observing if it triggers an attack, we can gain insights into the underlying mechanisms.^3^ This is also known as a human provocation model.^6^ These type of studies, along with others, have provided evidence that calcitonin gene-related peptide (CGRP), a neuropeptide found in abundance in the trigeminovascular system, plays a crucial role.^5^ When CGRP binds to its receptor, it activates a downstream signalling cascade involving cyclic adenosine monophosphate (cAMP).^5^ In a series of clinical trials, we have shown that administering CGRP or cilostazol, a phosphodiesterase (PDE)-3 inhibitor that prevents cAMP metabolization, can provoke migraine attacks in patients.^7-13^ This highlights the importance of CGRP and cAMP signalling in migraine pathogenesis. Assuming a single-cell system, it is plausible for a downstream mechanism, such as PDE3 inhibition by cilostazol, to influence an upstream mechanism like CGRP receptor activation through positive or negative feedback loops. Such interactions, where downstream components affect upstream components, are common in human biology and illustrate the complexity of intracellular signalling regulation. However, it is important to recognize that migraine pathogenesis is more intricate than a single-pathway single-cell system. The trigeminovascular system comprises multiple cells from different tissues, adding complexity to the signalling pathways and interactions involved.^3,5^ Clinical trials have demonstrated that administration of pituitary adenylate cyclase-activating polypeptide (PACAP) and vasoactive intestinal polypeptide (VIP) can also provoke migraine attacks in humans.^3,6,14^ Similar to CGRP, both PACAP and VIP are able to increase intracellular cAMP levels.^5,15,16^ Moreover, erenumab, a fully human immunoglobulin (Ig) G2 monoclonal antibody (mAb) that targets and blocks the CGRP receptor, was introduced as a migraine preventative drug in 2018.^17,18^ Other drugs targeting the CGRP signalling pathway have followed.^2^ However, not all patients benefit from these treatments. These observations suggest that migraine attacks may not solely depend on the CGRP receptor but could involve a unified downstream cAMP-mediated mechanism. However, this hypothesis has not been directly tested in humans.^3,5^

Thus, it is timely to ascertain whether migraine attack generation requires CGRP receptor activation. This can be established through the use of human provocation models. We hypothesized that treatment with erenumab would mitigate the physiological effects of administration of CGRP, but not those of cilostazol. To test this hypothesis, we conducted a randomized, double-blind, placebo-controlled, parallel trial.

## Materials and methods

The protocol was approved by the Regional Health Research Ethics Committee of the Capital Region of Denmark (identifier: H-19073983), the Danish Data Protection Agency (P-2020-652) and the Danish Medicines Agency (identifier: 2020033418). We obtained a signed consent form at the time of screening before any protocol-related procedures or assessments. All study-related procedures complied with the Declaration of Helsinki, with later revisions. The study is registered in ClinicalTrials.gov (identifier: NCT04452929).

### Participants

Adults aged 18–65 years old with a diagnosis of migraine according to the International Classification of Headache Disorders, Third Edition (ICHD-3),^4^ were eligible for participation if they had a history of migraine for ≥12 months prior to screening with ≥4 migraine days per month in the 3 months prior to screening. Key exclusion criteria were history of any other primary headache disorder except for tension-type headache, any secondary headache disorder, daily consumption of any medication other than oral contraception and prior treatment with monoclonal antibodies or participation in clinical trials with monoclonal antibodies during the preceding 12 months. Study eligibility was ensured by review of medical records and evaluation by headache specialists at the Danish Headache Center. A full overview of inclusion and exclusion criteria is available on ClinicalTrials.gov (identifier: NCT04452929).

### Study design and procedures

**Figure 1:**
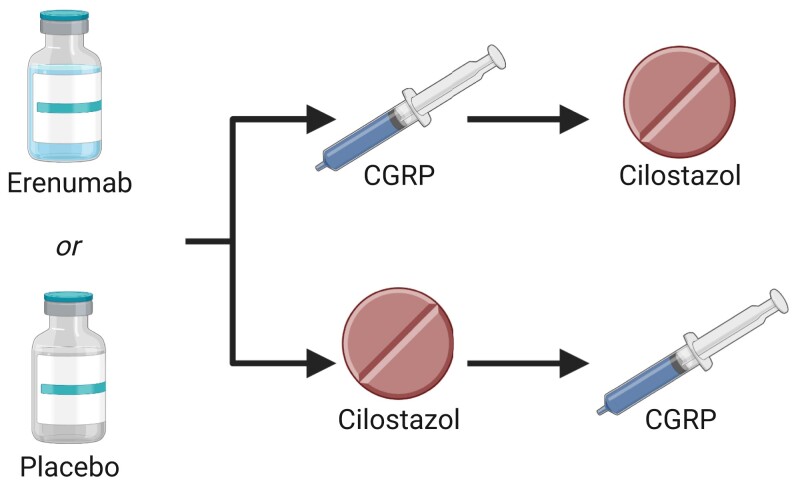
**Study design.** Participants were enrolled in a randomized, double-blind, placebo-controlled, parallel trial. Participants were randomly allocated to a subcutaneous administration of 140 mg of erenumab or placebo (isotonic saline). Seven to 21 days after study drug administration, participants were randomly allocated to receive a continuous intravenous infusion of 1.5 μg/min of calcitonin gene-related peptide (CGRP) over 20 min or oral intake of 200 mg cilostazol on separate experimental study days.

We enrolled participants in a randomized, double-blind, placebo-controlled, parallel trial at a single centre in Denmark (Fig. 1). Participants were randomly allocated to a subcutaneous administration of 140 mg of erenumab or placebo (isotonic saline). Erenumab was provided by Novartis Pharma AG and sorted in blinded packaging by Nomeco A/S. Independent pharmacy staff were responsible for randomization and allocation concealment. Seven to 21 days after study drug administration, participants were randomly allocated to receive a continuous intravenous infusion of 1.5 μg/min of CGRP over 20 min or oral intake of 200 mg cilostazol on two experimental study days separated by at least 3 days. Patients were informed that CGRP and cilostazol might induce headache or migraine attack with no additional information on induction frequency, time to onset or features; in addition, patients were informed that erenumab may mitigate the effects of these experimental triggers with no information provided on possible mechanism or site of action.

Patients arrived between 08:00 a.m. and 12:00 p.m. on the experimental study days. Patients were in a supine-position during study-related procedures and assessments. Patients were not allowed to consume any medication within 24 h or four times the plasma half-life of the drug (whichever longest) on the experimental study days except for oral contraception. Furthermore, to remain eligible, participants could not have had a migraine attack during the preceding 48 h nor a headache during the preceding 24 h prior to administration of the experimental trigger.

### Headache characteristics and diary

A headache specialist conducted a semi-structured interview at the screening visit. The interview included information on medical history, headache characteristics and frequency, family history of migraine and prior pharmacological treatment; these data were confirmed by review of medical records.

On the experimental study days, investigators recorded data on headache characteristics, vital signs and adverse events every 10 min for 90 min after administration of the experimental trigger. After the in-hospital phase, patients were discharged with a 12-h headache diary for hourly recordings of headache features, accompanying symptoms, intake of any rescue medication and adverse events.

### Haemodynamic variables

Patients rested in a quiet room for at least 30 min in a supine position before any measurements. During the in-hospital phase, we conducted electrocardiography and measured the blood pressure and heart rate. Furthermore, we used a high-resolution ultrasonography unit (Dermascan C; Cortex Technology: 20 MHz, bandwidth 5 MHz) as previously described to measure the diameter of the frontal branch of the superficial temporal artery and the radial artery.^19,20^

### Statistical considerations and analysis

Sample size calculations were based on the difference between two unpaired groups reporting migraine attacks. After administration of CGRP, we assumed that 51% of participants would report migraine attack in the placebo-treatment arm and 20% in the active-treatment arm. After administration of cilostazol, we assumed that 66% of participants would report migraine attack in the placebo-treatment arm and 20% in the active-treatment arm. These assumptions were based on previous migraine provocation studies, in which ∼66% of participants with migraine developed attacks after administration of CGRP and ∼86% after cilostazol,^7-13^ and that treatment prior to a provocation challenge lowers the expected induction rate by ∼20% (0.2 × historical induction rate).^21^ At 80% power and a 5% level of significance, we estimated that 72 patients would be needed in a two-arm parallel trial.

The primary end point was the incidence of migraine attacks in a 12-h observational period after administration of experimental triggers.^22^ The secondary end points were difference in area under the curve (AUC) values for headache intensity scores, superficial temporal artery diameter, radial artery diameter, heart rate and mean blood pressure. Pearson’s chi-squared test was used to analyse the incidence of migraine attacks and headache as binary categorical data. We made calculations of AUC values in accordance with the trapezium rule.^23^ We used the Mann–Whitney test to analyse differences in headache AUC scores after administration of CGRP or cilostazol between the active-treatment arm versus placebo-treatment arm. Analyses of haemodynamic AUC values were analysed using unpaired, two-way *t*-tests. All statistical analyses were performed using R (version 4.1.0).^24^ A 5% level of significance was accepted for all comparisons. We present data as mean values with standard deviation (SD), except the headache score which is presented as the median. Time to onset of migraine attack, time to intake of rescue medication, time to peak headache intensity and duration of migraine-associated symptoms are presented as median values. Baseline was defined as the time of the start of infusion (T_0_).

## Results

### Study population characteristics

**Figure 2:**
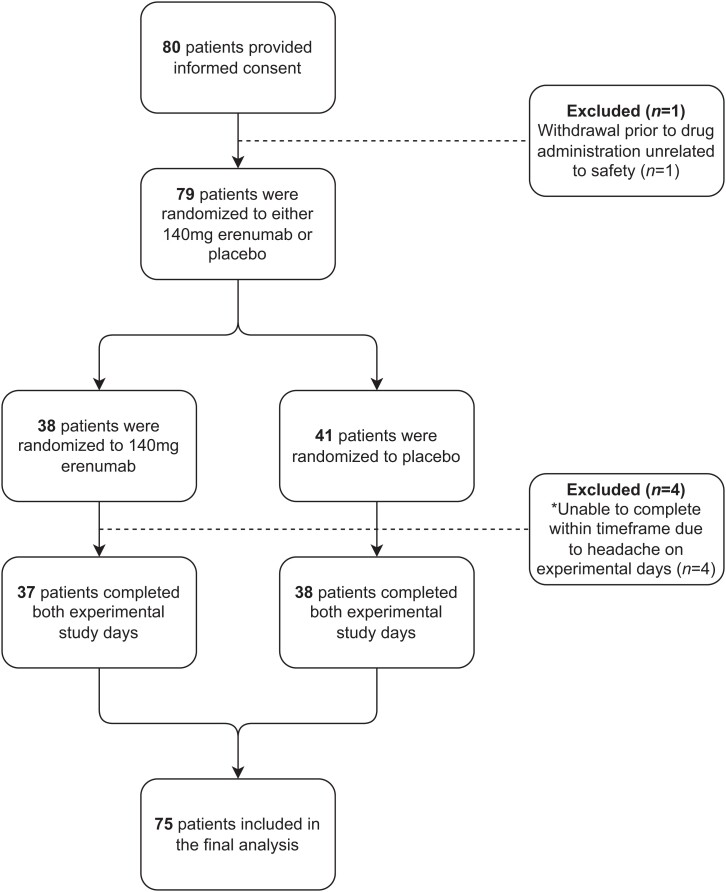
**Flow chart of participant recruitment of individuals with migraine**. To study the effects of calcitonin gene-related peptide (CGRP) receptor blockade on experimentally induced migraine attacks, we administered erenumab or placebo to adults with migraine in a randomized, double-blind, placebo-controlled, parallel trial design.

A total of 80 patients provided informed consent from July 2020 to July 2021; 75 patients completed both experimental study days and were included in the final analysis (Fig. 2). One patient discontinued study participation prior to administration of study drug for reasons unrelated to safety; four were not able to complete both experimental study days between Day 7 and Day 21 due to occurrence of headache within 24 h prior to administration of the experimental trigger. Demographic and baseline characteristics are presented in Table 1. Mean age was 33.4 years (SD: 11.1, range: 20–65 years), mean weight was 69.6 kg (SD: 12.8, range: 50–99 kg) and mean body mass index (BMI) was 23.9 (SD: 3.5, range: 19.1–34.8). For headache and medication intake frequency 3 months prior to enrollment, the mean number of monthly headache days was 10.6 days (SD: 4.7, range: 4–24 days), mean number of monthly migraine days was 7.2 days (SD: 3, range: 4–15 days) and mean number of monthly acute medication intake was 6.4 days (SD: 3.2, range: 0–14 days).

**Table 1:** Clinical characteristics of the study population

Baseline characteristics	All participants (*n* = 75)	Active group, 140 mg erenumab (*n* = 37)	Placebo group, saline solution (*n* = 38)
Age, mean (SD; range)	33.4 (11.1; 20–65)	35.5 (11; 21–56)	31.3 (10.7; 20–65)
BMI, mean (SD; range)	23.9 (3.5; 19.1–34.8)	23 (3.2; 19.1–34.7)	24.7 (3.6; 19.3–34.8)
Weight, mean (SD; range)	69.6 (12.8; 50–99)	68.3 (12.4; 50–99)	70.8 (13.1; 55–99)
Male/female, *n* (%)	9; 66 (12%; 88%)	5; 32 (14%; 86%)	4; 34 (11%; 89%)
Diagnosed with migraine without aura, *n* (%)	56 (75%)	27 (73%)	29 (76%)
Diagnosed with migraine with aura, *n* (%)	7 (9%)	4 (11%)	3 (8%)
Diagnosed with both migraine without aura and migraine with aura, *n* (%)	12 (16%)	6 (16%)	6 (16%)
Chronic migraine, *n* (%)	11 (15%)	3 (8%)	8 (21%)
High frequency episodic migraine^a^, *n* (%)	19 (25%)	11 (30%)	8 (21%)
Family history of migraine, *n* (%)	49 (65%)	28 (76%)	21 (55%)
Number of monthly headache days^b^, mean (SD; range)	10.6 (4.7; 4–24)	9.6 (4; 4–19)	11.5 (5.1; 4–24)
Number of monthly migraine days^b^, mean (SD; range)	7.2 (3; 4–15)	7.1 (3.1; 4–15)	7.2 (3; 4–15)
Number of monthly acute medication intake days^b^, mean (SD; range)	6.4 (3.2; 0–14)	6.4 (2.8; 0–12)	6.5 (3.6; 0–14)
Number of prior failed preventive medications, median (range)	1 (0–7)	1 (0–6)	1 (0–7)

BMI = body mass index; SD = standard deviation.

^a^High frequency episodic migraine defined as <15 monthly headache days with ≥8 monthly migraine days.

^b^Data were collected retrospectively at baseline through a semi-structured interview.

### Intracellular mechanisms can induce migraine attacks independent of CGRP receptor activation

**Figure 3:**
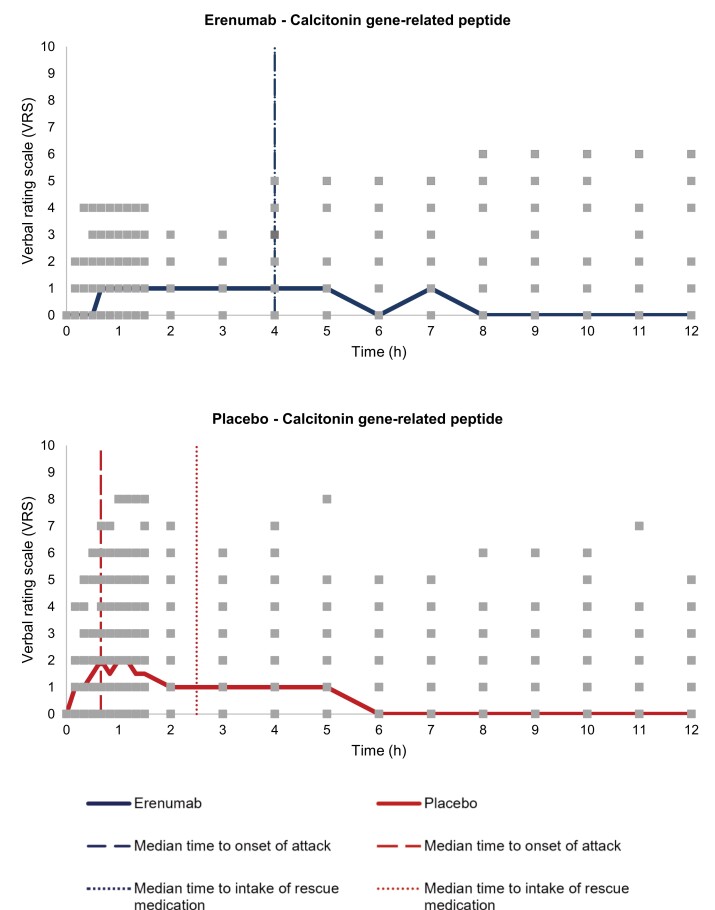
**Headache characteristics after administration of calcitonin gene-related peptide (CGRP) in individuals with migraine randomized to erenumab or placebo**. Participants were randomized to erenumab or placebo 7–21 days prior to administration of CGRP. Headache intensity was rated on an 11-point numeric rating scale from 0 to 10 (median, thick lines). The median time to onset of migraine attack was 240 min (range: 30–540 min) in the erenumab group and 40 min (range: 10–480 min) in the placebo group. The median time to intake of rescue medication was 240 min (range: 120–660 min) in the erenumab group and 150 min (range: 40–480 min) in the placebo group. Square markers represent individual reported headache intensity.

We demonstrated that erenumab can alleviate migraine attack induction with CGRP with 10 of 37 [27% (95% CI, 13–41)] participants developing migraine attacks compared to 20 out of 38 [53% (95% CI, 37–69)] participants who received placebo (*P* = 0.024) (Fig. 3 and Table 2). The incidence of headache following intravenous infusion of CGRP was significantly reduced in the erenumab group compared to the placebo group [24 (65%) versus 35 (92%), *P* = 0.004]. The most common localization of headache in the active-treatment group was in frontal region [19 (51%)] followed by temporal [14 (38%)], occipital [11 (30%)] and parietal [7 (19%)] regions. In the placebo-treatment group, the most common localization was frontal region [30 (81%)] followed by temporal [25 (66%)], occipital [15 (41%)] and parietal [5 (13%)] regions. We did not find a difference in the area under the curve (AUC) for headache intensity between the erenumab group and the placebo group following intravenous infusion of CGRP (AUC_0–12h_, *P* = 0.180). The incidence of migraine-associated symptoms in the active-treatment group compared to the placebo-treatment group was, for nausea, 9 (24%) and 13 (34%), photophobia, 16 (43%) and 23 (61%), and phonophobia, 6 (16%) and 14 (37%), respectively. The median time to onset in the active-treatment group compared to the placebo-treatment group was, for nausea, 120 min (range, 10–660 min) and 30 min (range, 10–120 min), photophobia, 55 min (range, 10–420 min) and 30 min (range, 10–360 min), and phonophobia, 210 min (range, 10–42 min) and 40 min (range, 10–180 min), respectively. The median time of duration in the active-treatment group compared to the placebo-treatment group was, for nausea, 120 min (range, 60–630 min) and 80 min (range, 10–640 min), photophobia, 175 min (range, 20–590 min) and 190 min (range, 10–710 min), and phonophobia, 120 min (range, 10–360 min) and 235 min (range, 20–700 min), respectively.

**Table 2:** Migraine attack induction rate

	Erenumab (*n* = 37)	Placebo (*n* = 38)
Migraine attack following CGRP administration	27% (*n* = 10; 95% CI, 13–41%)	53% (*n* = 20; 95% CI, 37–69%)
Migraine attack following cilostazol administration	76% (*n* = 28; 95% CI, 62–90%)	82% (*n* = 31; 95% CI, 69–94%)
Interpretation	Erenumab mitigates the physiological effects of CGRP, but not cilostazol, in adults with migraine.	

Calcitonin gene-related peptide (CGRP): There was a significant difference in the incidence of migraine attacks following administration of CGRP in individuals with migraine randomized to erenumab compared to placebo (*P* = 0.024). Cilostazol: There was no difference in the incidence of migraine attacks following administration of cilostazol in individuals with migraine randomized to erenumab compared to placebo (*P* = 0.533).

**Figure 4:**
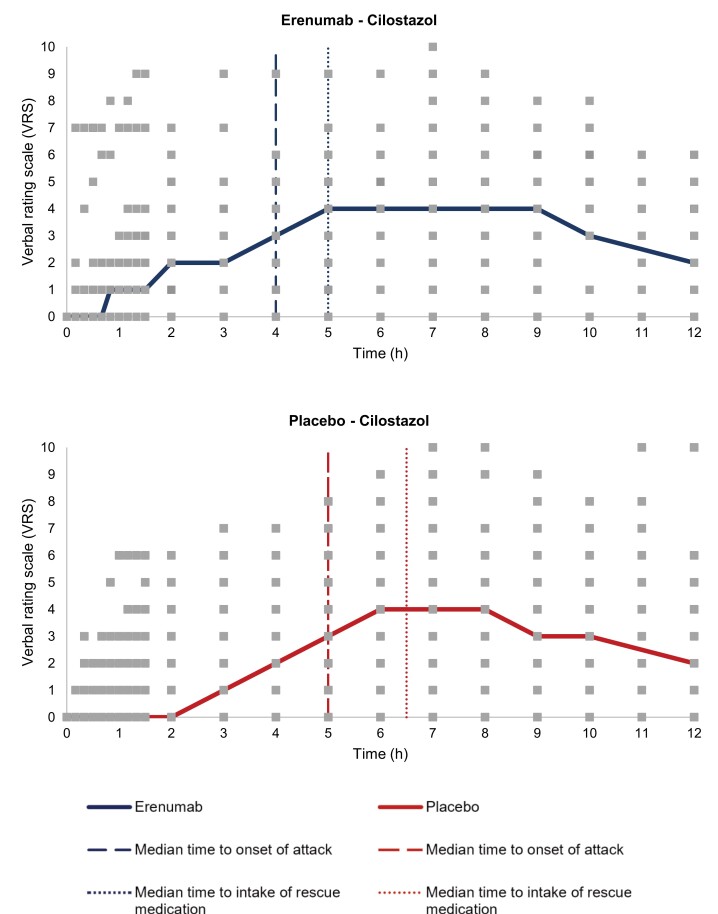
**Headache characteristics after administration of cilostazol in individuals with migraine randomized to erenumab or placebo.** Participants were randomized to erenumab or placebo 7–21 days prior to administration of cilostazol. Headache intensity was rated on an 11-point numeric rating scale from 0 to 10 (median, thick lines). The median time to onset of migraine attack was 240 min (range: 10–600 min) in the erenumab group and 300 min (range: 20–600 min) in the placebo group. The median time to intake of rescue medication was 300 min (range: 120–720 min) in the erenumab group and 390 min (range: 70–600 min) in the placebo group. Square markers represent individual reported headache intensity.

We found no difference in cilostazol-induced migraine attacks between participants who received erenumab versus placebo. Cilostazol-induced migraine attacks in 28 of 37 [76% (95% CI, 62–90)] participants in the erenumab group compared to 31 out of 38 [82% (95% CI, 69–94)] participants in the placebo group (*P* = 0.533) (Fig. 4 and Table 2). In the placebo group, all participants (except one) who had a migraine attack following a CGRP challenge also had a migraine attack following a cilostazol challenge. In the erenumab group, all participants who had a migraine attack following a CGRP challenge also had a migraine attack following a cilostazol challenge. Erenumab did not reduce the incidence of headache after intake of cilostazol [33 (89%) versus 37 (97%), *P* = 0.156]. The most common localization of headache in the active-treatment group was in frontal region [24 (65%)], followed by temporal [20 (53%)], occipital [14 (38%)] and parietal [14 (38%)] regions. In the placebo-treatment group, the most common localization was frontal region [30 (79%)], followed by temporal [29 (76%)], occipital [14 (37%)] and parietal [8 (21%)] regions. There was no difference in AUC for headache intensity (AUC_0–12h_, *P* = 0.795).

The incidence of migraine-associated symptoms in the active-treatment group compared to the placebo-treatment group was, for nausea, 17 (46%) and 22 (58%), photophobia, 24 (65%) and 23 (61%), and phonophobia, 17 (46%) and 19 (50%), respectively. The median time to onset in the active-treatment group compared to the placebo-treatment group was, for nausea, 240 min (range, 10–660 min) and 300 min (range, 10–660 min), photophobia, 310 min (range, 10–420 min) and 240 min (range, 10–540 min), and phonophobia, 180 min (range, 10–420 min) and 240 min (range, 20–540 min), respectively; the median time of duration in the active-treatment group compared to the placebo-treatment group was, for nausea, 210 min (range, 60–540 min) and 150 min (range, 30–340 min), photophobia, 300 min (range, 50–710 min) and 420 min (range, 30–710 min), and phonophobia, 315 min (range, 30–640 min) and 240 min (range, 50–600 min), respectively.

### CGRP-mediated vasodilation is mitigated by a blockade of the CGRP receptor

**Figure 5:**
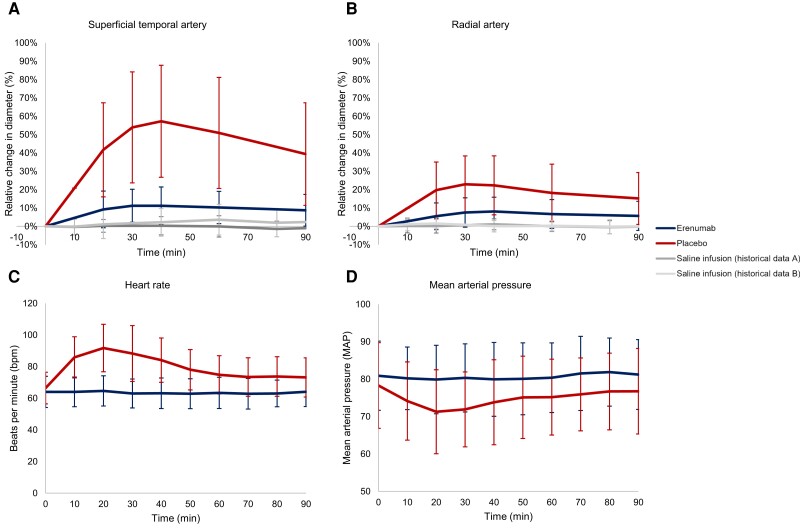
**Haemodynamic changes following administration of calcitonin gene-related peptide (CGRP) in individuals with migraine randomized to erenumab or placebo**. (**A**) Mean change in diameter of the superficial temporal artery relative to baseline. The increase in the diameter of the superficial temporal artery in individuals randomized to erenumab was significantly less compared to placebo (*P* < 0.00001). Erenumab, however, did not completely inhibit the increase in diameter as there was a small increase compared to historical data with saline infusion in individuals with migraine without preventive medication. (**B**) Mean change in diameter of the radial artery relative to baseline. The increase in the diameter of the radial artery in individuals randomized to erenumab was significantly less compared to placebo (*P* < 0.00001). Erenumab, however, did not appear to completely inhibit the increase in diameter as there was a small increase compared to historical data with saline infusion in individuals with migraine without preventive medication. (**C**) Mean heart rate in absolute values. There was a significant increase in the heart rate in individuals randomized to placebo compared to erenumab (*P* < 0.00001). (**D**) Mean arterial pressure in absolute values. There was a significant decrease in the mean arterial pressure in individuals randomized to placebo compared to erenumab (*P* < 0.01). Error bars represent standard deviation. Historical data, A^33^ and B.^35^

**Figure 6:**
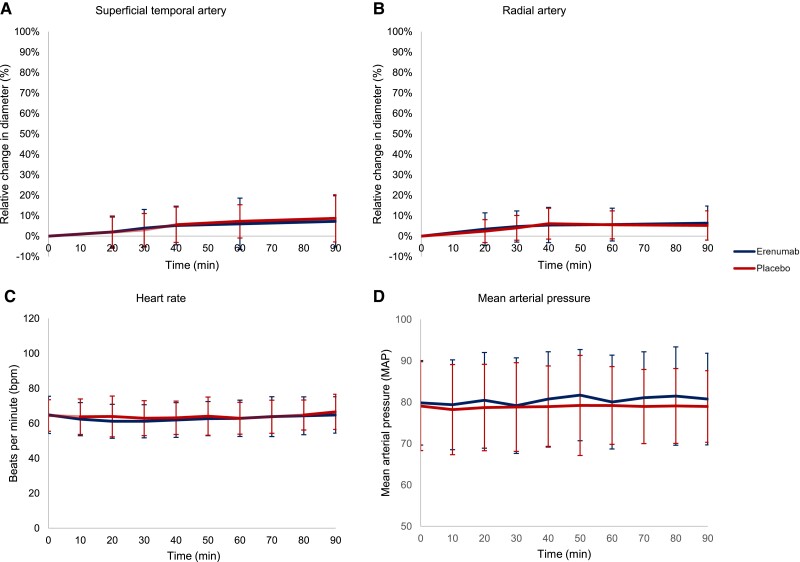
**Haemodynamic changes following administration of cilostazol in individuals with migraine randomized to erenumab or placebo**. (**A**) Mean change in diameter of the superficial temporal artery relative to baseline. There was no difference in the diameter of the superficial temporal artery in individuals randomized to erenumab compared to placebo (*P* = 0.238). (**B**) Mean change in diameter of the radial artery relative to baseline. There was no difference in the diameter of the radial artery in individuals randomized to erenumab compared to placebo (*P* = 0.984). (**C**) Mean heart rate in absolute values. There was no difference in the heart rate in individuals randomized to erenumab compared to placebo (*P* = 0.659). (**D**) Mean arterial pressure in absolute values. There was no difference in the mean arterial pressure in individuals randomized to placebo compared to erenumab (*P* = 0.480). Error bars represent standard deviation.

We found that erenumab significantly attenuated CGRP-induced dilation of the superficial temporal artery (AUC_0–90min_, *P <* 0.00001) and the radial artery (AUC_0–90min_, *P <* 0.00001) compared to placebo (Fig. 5). Furthermore, erenumab significantly attenuated CGRP-induced changes in mean arterial blood pressure and in heart rate (mean arterial blood pressure AUC_0–90min_, *P* < 0.01; heart rate AUC_0–90min_, *P* < 0.00001) (Fig. 5). Following intake of cilostazol, there was no difference in the diameter of the superficial temporal artery (*P* = 0.238) or radial artery (*P* = 0.984) in individuals randomized to erenumab compared to placebo (Fig. 6). Furthermore, there were no differences between erenumab and placebo for other haemodynamic variables (mean arterial blood pressure AUC_0–90min_, *P* = 0.480; heart rate AUC_0–90min_, *P* = 0.659) (Fig. 6).

### Adverse events

The proportion of patients reporting warm sensations, palpitations and flushing in the erenumab group was lower than in the placebo group (Table 3).

**Table 3:** Adverse events

	CGRP		Cilostazol	
	Erenumab (*n* = 37)	Placebo (*n* = 38)	Erenumab (*n* = 37)	Placebo (*n* = 38)
Abdominal discomfort	0 (0%)	2 (5%)	1 (3%)	1 (3%)
Cold sensations	0 (0%)	0 (0%)	1 (3%)	1 (3%)
Dizziness	1 (3%)	1 (3%)	2 (5%)	2 (5%)
Dry eyes	1 (3%)	4 (11%)	0 (0%)	0 (0%)
Ear fullness	0 (0%)	2 (5%)	0 (0%)	0 (0%)
Flushing	4 (11%)	34 (89%)	0 (0%)	0 (0%)
Itch	0 (0%)	1 (3%)	0 (0%)	0 (0%)
Palpitations	6 (16%)	31 (82%)	9 (24%)	6 (16%)
Warm sensations	15 (41%)	37 (97%)	15 (41%)	13 (34%)

Reports of adverse events following administration of calcitonin gene-related peptide (CGRP) and cilostazol in individuals with migraine randomized to erenumab or placebo.

Warm sensations and palpitations are common adverse events after intake of cilostazol in individuals with migraine.^11-13^ Following administration of cilostazol, the proportion of patients reporting warm sensations and palpitations in the erenumab group was similar to the placebo group (Table 3).

## Discussion

A novel finding of our study is that migraine attacks induced by upregulation of intracellular cAMP levels are not affected by blocking the CGRP receptor. Our work provides clinical evidence that cAMP-evoked migraine attacks do not require CGRP receptor activation. We want to highlight three important observations in this context: (i) patients who were pretreated with erenumab still experienced migraine attacks when cilostazol was administered; (ii) patients who were susceptible to migraine attacks following CGRP administration also had migraine attacks induced by cilostazol; and (iii) most participants who did not experience migraine attacks after receiving CGRP did have migraine attacks when cilostazol was administered.

Cilostazol, a PDE-3-inhibitor, blocks both isoforms of PDE3, which is responsible for metabolizing cAMP.^25^ As a result, cilostazol leads to accumulation of intracellular cAMP. It is important to note that cilostazol does not directly increase cAMP generation but inhibits its degradation, thus relying on upstream regulators such as the CGRP receptor or other factors. Notably, the observation that cilostazol induces migraine attacks even in the presence of a CGRP receptor blockade suggests the existence of a CGRP-independent pathway for migraine attacks in humans. A preclinical study demonstrated that PACAP-induced hypersensitivity in a mouse model of migraine-like pain is independent of CGRP.^26^ However, it is important to interpret these findings cautiously as CGRP receptor antagonism was still able to inhibit pain induced by cilostazol in the same mouse model.^27^ This suggests that the translatability of the model may have limitations. Nonetheless, the PACAP signalling pathway is of interest as both the ligand and its receptors are expressed within the trigeminovascular system.^5^ In humans, PACAP binds to three different receptors (VPAC1, VPAC2 and PAC1), and experimental data have shown that activation of these receptors can increase intracellular levels of cAMP.^5^ Furthermore, a recent clinical trial demonstrated that Lu AG09222, an anti-PACAP mAb, was effective in inhibiting PACAP38-induced cephalic vasodilation and reducing concomitant headache in healthy volunteers.^28^

Cilostazol primarily inhibits the isoform PDE3A, which is predominantly found in vascular smooth muscle cells.^29^ Its inhibition of PDE3 leads to dilation cranial arteries in both healthy volunteers and individuals with migraine.^30,31^ Of note, cilostazol is used to relieve symptoms of intermittent claudication. Partly because of its vasodilatory effect.^32^ Our findings support a vascular site of action, as we observed a minor increase in arterial diameter during the 90 min in-hospital monitoring phase. However, further observation over a longer period is necessary to confirm these results, as previous studies with longer observational periods have shown a more pronounced increase in arterial diameter over time.^30,31^ These results align with the observation that stimulation of downstream targets of cilostazol, such as ATP-sensitive potassium (K_ATP_) channels and large-conductance calcium-activated potassium (BK_Ca_) channels, can induce migraine attacks in parallel with vasodilation in individuals with migraine.^33-35^ Interestingly, cilostazol can bind directly to the inner surface of BK_Ca_ channels, stimulating their activity.^36^ Sumatriptan (an anti-migraine drug) inhibits cAMP/PKA-signalling^37-40^ and constricts the superficial temporal artery and middle meningeal artery in parallel with pain relief during cilostazol-induced migraine attacks. This suggests a perivascular or peripheral site of action.^30^ Given that cilostazol is a lipophilic compound,^41,42^ we cannot exclude additional peripheral or central neuronal sites of action. Although both isoforms of PDE3 are co-localized with CGRP in the trigeminal ganglion,^43^ PDE3 activity does not regulate CGRP release.^27^ However, we cannot exclude a direct effect of cilostazol on the trigeminal ganglion as the PDE3A isoform is present in non-neuronal cells there.^44^ Furthermore, activation of the cAMP-PKA second messenger pathway can sensitize dural mechanonociceptors in rats, potentially independent of a vascular involvement. However, further data are needed to elucidate the potential effects of cilostazol within the nervous system, considering its limited ability to cross the blood–brain barrier in several species,^45^ which is consistent with the rarity of adverse events related to the CNS for cilostazol.^32^

We found that CGRP receptor blockade did not completely prevent CGRP-induced migraine attacks. One-quarter of patients in the erenumab group still experienced a migraine attack following administration of CGRP, which exceeds the expected rate of a nocebo response [8.1% (95% CI, 2.5–15.5%)].^46^ This finding is clinically interesting as one-third of migraine patients do not report a substantial reduction in migraine frequency after treatment with an anti-CGRP receptor mAbs.^18,47,48^ It is worth mentioning that CGRP receptor blockade did partially mitigate CGRP-induced vasodilation, but there was still a residual vascular response. This suggests that CGRP signalling, although to a lesser extent, may also occur through receptors other than the canonical CGRP receptor. This residual effect could provide a partial explanation for why breakthrough migraine attacks can still occur despite treatment with anti-CGRP receptor mAbs, albeit at a lower frequency. It is possible that in patients treated with a CGRP receptor antagonist, residual CGRP receptors may still exert a minor physiological effect.^49-53^ Supposedly, circulating CGRP could bind to the AMY1 receptor, leading to mild vasodilation,^49-53^ as observed in our study. Interestingly, other peptides in the calcitonin family that share receptor affinity have also been implicated in migraine pathogenesis.^49-51^ Further data are needed to elucidate the role of the AMY1 receptor and its interaction with CGRP signalling in migraine pathophysiology.

Although we applied rigorous criteria during recruitment and standardized experimental procedures, we could not rule out various environmental factors (e.g. stress, food intake) after discharge from the laboratory and the subsequent outpatient 12-h observation phase. However, alternative 12-h inpatient monitoring would have limited the feasibility of the study. In addition, the migraine attack induction rates for CGRP and cilostazol in the placebo arm were comparable to previously reported studies with these substances.^11,12,31,54^ Differences in T_max_ between participants are possible after oral administration of cilostazol. Nonetheless, the induction rate and median time to onset of cilostazol-induced migraine attacks is comparable to previous reports.^11,12,31,54^ As mentioned earlier, we noticed a slight increase in the diameter of the superficial temporary artery in both study arms after administering cilostazol. However, a longer observation period would have given us more information about potential vascular effects, as previous studies have shown a progressive increase in arterial diameter over time.^30,31^ We cannot exclude differences in bioavailability of erenumab between individuals. Nevertheless, mean T_max_ is 5.5 days in healthy volunteers and consistent with an early onset of effect within the first week of treatment in patients.^55-57^ Experimental study days took place between Day 7 and Day 21 after randomization, enabling onset and maintenance of efficacy.

## Conclusions

The present data provide further evidence for the crucial role of second messenger signalling in a migraine attack. We demonstrate that migraine attack generation does not require CGRP receptor activation. These findings highlight the relevance of targeting downstream signalling pathways or other molecules involved in cAMP signalling, such as PACAP, as potential therapeutic targets for the treatment of migraine.

## Data Availability

Anonymized datasets generated and/or analysed during the current study are available upon reasonable request and following the acquisition of necessary permissions.
